# Cytotoxic effects of *Pseudocerastes persicus* venom and its HPLC fractions on lung cancer cells

**DOI:** 10.1590/1678-9199-JVATITD-2019-0009

**Published:** 2019-09-16

**Authors:** Benyamin Shahbazi, Zahra Salehi Najafabadi, Hamidreza Goudarzi, Mahnaz Sajadi, Fatemeh Tahoori, Masoumeh Bagheri

**Affiliations:** 1 Payame Noor University of Tehran Shargh, Tehran, Iran.; 2 Razi Vaccine and Serum Research Institute, Agricultural Research, Education and Extension Organization, Karaj, Iran.; 3 Tofigh Daru Research and Engineering Company, Tehran, Iran.

**Keywords:** *Pseudocerastes persicus* venom, Persian horned viper, HPLC fractions, Cytotoxicity, Apoptosis

## Abstract

**Background:**

Several studies have pointed out that certain snake venoms contain compounds presenting cytotoxic activities that selectively interfere with cancer cell metabolism. In this study, *Pseudocerastes persicus* venom and its fractions were investigated for their anticancer potential on lung cancer cells.

**Methods:**

Lung cancer cells (A549) and normal fibroblast cells (Hu02) were treated with the *P. persicus* venom and its HPLC fractions and the cell cytotoxic effects were analyzed using MTT and lactate dehydrogenase release assays. Apoptosis was determined in venom-treated cell cultures using caspase-3 and caspase-9 assay kits.

**Results:**

The treatment of cells with HPLC fraction 21 (25-35 kDa) of *P. persicus* venom resulted in high LDH release in normal fibroblast cells and high caspase-3 and caspase-9 activities in lung cancer cells. These results indicate that fraction 21 induces apoptosis in cancer cells, whereas necrosis is predominantly caused by cell death in the normal cells. Fraction 21 at the final concentration of 10 μg/mL killed approximately 60% of lung cancer cells, while in normal fibroblast cells very low cell cytotoxic effect was observed.

**Conclusion:**

HPLC fraction 21 at low concentrations displayed promising anticancer properties with apoptosis induction in the lung cancer cells. This fraction may, therefore, be considered a promising candidate for further studies.

## Background

Snake venom is a highly complex mixture of organic and inorganic compounds that include peptides, enzymes, low-molecular mass proteins that have specific chemical and biological activities and non-protein inclusions [1,2]. There are many venom-derived drugs on the market against different types of human diseases, some examples are captopril and enalapril against hypertension, ziconotide for management of severe chronic pain and batroxobin for acute cerebral infarction [3,4].

Undoubtedly, cancer is one of the primary causes of human deaths worldwide [5,6]. It can be treated with surgery, chemotherapy and/or radiation, targeted therapy, immunotherapy and even nonselective cytotoxic drugs [7]. Therefore, the investigation and discovery of new drugs for treatment of cancer are the objectives of research in biotechnology [8,9]. Numerous studies, in phase I and phase II of clinical trials, using advanced proteomics and genomics approaches described that venom peptides can induce cytotoxic effects and apoptosis on cancer cell lines are [3,10]. Cytotoxins are one of the most important toxins isolated from various snake venoms and they alter the cellular metabolism through interaction with specific cellular receptors, damaging the cell membranes or blocking the ion channels or the signal transduction pathways [11]. Ion channels targeting cancer cells include polycystin complexes[12], chloride channels, sodium channels and potassium channels [3,10]. 

The enzymes and proteins with promising anticancer activities include phospholipases A_2_ (cytotoxicity), L-amino acid oxidases (LAAOs - apoptosis), metalloproteinases (inhibitor of cell proliferation), peptides such as cardiotoxin III (anti-angiogenic) and cytotoxin P4 (cytotoxicity), cytotoxins CT1, CT2 and CT3 (cytotoxicity), lectins (cytotoxicity), disintegrins (anti-angiogenic), serineproteases such as ancrod (inhibitor of tumor growth) etc. [1,2]. 

The Persian horned viper *Pseudocerastes persicus* is a venomous viper species found in the Middle East and Asia. *P. persicus* venom exhibits strong hemorrhagic activity and is potently coagulopathic. Although there are some research on *P. persicus* venom composition, currently little information about specific activities of the venom is available.

In the present study, we investigated the cytotoxic induction capacity of *P. persicus* venom and its fractions on lung cancer cells and normal fibroblast cells *in vitro*. 

## Methods

## Materials and chemicals

Dimethylsulfoxide (DMSO), Dulbecco’s modified Eagle’s medium (DMEM), penicillin-streptomycin (Pen-Strep), L-glutamine, fetal bovine serum (FBS), 3-(4, 5 dimethylthiazol-2-yl)-2,5-diphenyl-tetrazolium bromide (MTT), Bradford reagent and sodium bicarbonate were purchased from Sigma. Caspase-3 and caspase-9 assay kits (Colorimetric) were purchased from Abcam, USA.

## Venom preparation

Freeze-dried *P. persicus* snake venom was obtained from the department of venomous animals and antivenom production of Razi Vaccine and Serum Research Institute. Lyophilized venom was dissolved in sterile double-distilled water. After centrifugation at 4000 RCF for 20 min at 4°C, the supernatant was passed through 0.45 μm nitrocellulose filter (MilliporeSigma, USA) and the protein concentration was examined by Bradford method.

## Cell lines and cell culture

Lung cancer cells (A549) and normal fibroblast cells (Hu02) were purchased from Iranian Biological Resource Center. Above cells were cultured in T75 cell culture flask containing DMEM medium, 10% FBS, 1% Pen-Strep, 2 mM L-glutamine. Cells were subcultivated using trypsin-EDTA (0.05% trypsin) in 96-well plates (Corning, USA) at a density of 15000 cells/well in 100 μL complete medium. All cells were incubated overnight at 37°C with 5% CO_2_ in a humidified incubator. 

## *In vitro* cytotoxicity assay

Cytotoxicity of the *P. persicus* venom was examined by colorimetric MTT assay [13,14]. The cells were subcultivated in three 96-well plates as mentioned before. Following overnight incubation, fresh complete medium with different concentrations of venom (0, 1, 3, 5, 10, 15, 18, 20, 30, 50 µg/mL) were added to the wells in triplicate. The cells were harvested after treatment of 24 and 48 hours. 

The culture media was removed and the wells were washed by adding 100 μL of 1× PBS buffer per well and the PBS was immediately removed. Then, 100 μL of DMEM medium without FBS and 20 μL MTT (5 mg/mL) was added to each well and the plates were incubated at 37°C in the dark for two hours. MTT is a tetrazolium dye that is reduced by specific mitochondrial enzymes (in the live cells) to formazan, an insoluble crystalline product. After incubation, the wells were washed again with PBS buffer. One hundred microliters of DMSO was added to each well and the plate was shaken for 5 min in the dark in order to dissolve the formazan crystals to deep purple color. Thirty microliters of glycine buffer (0.1 M glycine, 0.1 M NaCl, pH 10.5) was then added to each well and the absorbance was measured at 570 nm using a plate reading spectrophotometer (Bio-Rad Laboratories, USA). This assay was also employed to assess the half-maximal inhibitory concentration value (IC_50_) of the venom in each cell line.

## Assay to measure caspase-3 and caspase-9 activities

The activities of caspase-3 and caspase-9 were evaluated using the colorometric Caspase-3 (#ab39401, Abcam, USA) and Caspase-9 (#ab65608, Abcam, USA) assay kits according to manufacturer’s instructions. In brief, the cells were cultured in T25 flasks and were treated with IC_50_ concentrations of crude venom and they were collected at 12, 24 and 48 hours. 

The cells were harvested and homogenized in lysis buffer. After that, 200 μg of each lysate was mixed with 2× reaction buffer, DTT and substrate. The mixture was incubated at 37°C for two hours. Substrate cleavage was measured using a spectrophotometer at 400 or 405 nm. The caspase activity in an apoptotic sample increases due to cleavage product, comparing an uninduced control. 

## Lactate dehydrogenase release assay

Lactate dehydrogenase (LDH) is an enzyme that is released into extracellular space when the plasma membrane is damaged. Therefore, measuring the amount of LDH release is a useful assay to discriminate between apoptosis and necrosis. 

Hu02 and A549 cells were cultured in a 96-well plate and were treated in triplicates with 5, 10, 20, 30 µg/mL HPLC fraction 21 for 24 hours. LDH activity was measured in 100 µL of media overlaying the cells using LDH commercial kit (Pars Azmun, Iran) according to manufacturer’s protocol. In order to measure the cytosolic LDH, the cells in the wells were lysed with 100 µL of 1% (v/v) Triton X-100 and the cell lysates were assayed for LDH activity.

$$\mathrm{Percentage of released LDH}\;=\frac{\mathrm{Released LDH (in the medium)}\;}{\mathrm{Total LDH (in the medium and cell lysates)}}$$

## Venom fractionation by RP-HPLC

The lyophilized venom sample was dissolved in the sterile double-distilled water to achieve a final concentration of 5 mg/mL and injected into a Waters C18 reversed phase HPLC column (100Å, 5 µm, 4.6 × 150 mm) equilibrated with solvent A (water/0.1% TFA) at room temperature. The component of the venom was eluted at flow rate of 1 mL/min with increasing the concentration of solvent B (acetonitrile/0.1% TFA) using a step gradient program in 65 minutes. The absorbance of each component was monitored at 214 nm and the peaks were collected manually once identified by the detector. The fractions were lyophilized and reconstituted in DMEM medium and their cytotoxicity was analyzed as mentioned in the previous section. 

## SDS-PAGE analysis

SDS-PAGE analysis of crude venom and cytotoxic chromatographic fractions was performed under denaturating conditions using 12% polyacrylamide gel in the presence of DTT, according to the method described by Laemmli [15]. Protein bands were visualized by silver staining. 

## Morphological studies

Cells were subcultivated in a 96-well plate in complete DMEM medium and incubated at 37°C overnight. Subsequently, the cells were treated with different concentrations of venom for 24 hours and their morphological characterization was carried out using an inverted phase contrast microscope (Labomed TCM 400, USA). 

## Data analysis

IC_50_ was calculated using a dose-response curve. The difference between each experiment group and the control group was measured using an independent t-test. Values were presented as means ± standard deviation (n = 3). Statistical significance is indicated as follows: *p ≤ 0.05, **p ≤ 0.01, and ***p ≤ 0.001.

## Results

## Effect of *P. persicus* crude venom on morphology of normal fibroblast and lung cancer cells

**Figure 1.**
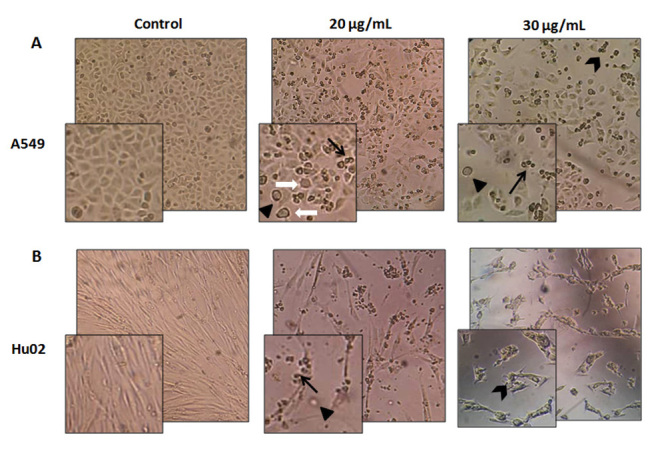
Morphological and cell growth assessment of (**A**) A549 and (**B**) Hu02 cells treated with *P. persicus* crude venom. Morphological differences in the cells were observed under Labomed TCM 400 inverted phase contrast microscope (magnification 100×) after treatment with 20 and 30 µg/mL of *P. persicus* crude venom for 24 hours. Control shows the untreated cells. Irregular shape of the cells (A549 normal cells), cell rounding (filled triangles), cellular debris (arrowheads), apoptotic bodies (black arrows) and cytoplasmic blebbing (white arrows) are observable in the venom treated cells

Lung cancer A549 cells and normal fibroblast Hu02 cells were used for evaluation of the cytotoxic effects of *P. persicus* venom. Figure 1 shows the morphological changes of A549 and Hu02 cells 24 hours after incubation with 20 µg/mL and 30 µg/mL of the crude venom. Phase contrast images of untreated A549 cells exhibited regular polygonal cells with clearly define edge. After 24 hours of incubation with 20 µg/mL of venom, morphological abnormalities such as rounded cells, apoptotic bodies and loss of adhesion were observed in the cells (Figure 1A). These changes were associated with apoptosis in A549 cells. Untreated Hu02 cells exhibited a thin and elongated shape. Twenty-four hours after venom exposition, morphological alterations including membrane disruption, production of cellular debris and apoptotic bodies were observed in the Hu02 cells (Figure 1B and C). Observations 48 hours after treatment revealed an increased number of dead cells (data not shown). 

Treatment of cells with higher doses of venom (30 μg/mL) caused growth inhibition and appearance of large areas devoid of cells. In addition, cells were likely to detach from the surface and float on the medium. 

## Cytotoxicity of *P. persicus* crude venom on lung cancer cells *versus* normal fibroblast cells

**Figure 2.**
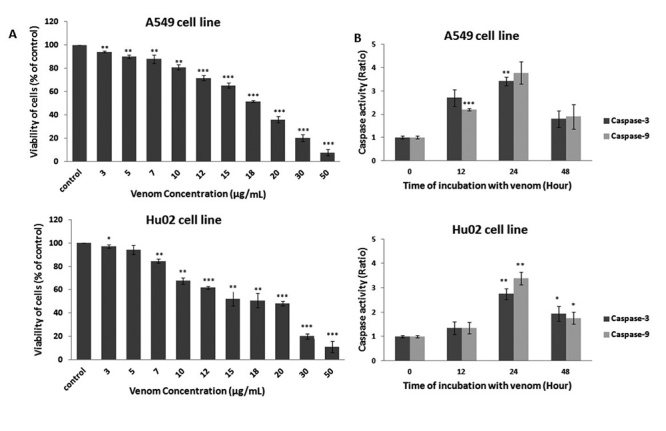
Effects of *P. persicus* venom on cell viability and caspase-3 and caspase-9 activities compared to untreated cell. (**A**) Cytotoxic activity of *P. persicus* crude venom on A549 and Hu02 cells after 24 hours of treatment with different venom concentrations. The viability of cells was estimated by MTT assay. (**B**) Activities of caspase-3 and caspase-9 enzymes in A549 and Hu02 cells after venom treatment. The cell lines were treated with IC50 concentrations of *P. persicus* venom for 12, 24 and 48 hours and harvested separately. Activities of caspases were determined by substrate cleavage assay. All data were presented as mean ± S.D (n = 3). Statistical significance versus control (*p < 0.05; **p < 0.01; ***p < 0.001).

Treatment of two cell lines with *P. persicus* crude venom at 3-50 µg/mL concentrations for 24 hours significantly decreased cell viability dose- and time-dependent manner, compared with control cells (Figure 2A). It is interesting that using the highest venom dose (50 µg/mL) or longer incubation period (48 hours), the cells were killed, supposedly by acute necrosis and no apoptotic bodies were observed (data not shown).

The IC_50_ for A549 and Hu02 cells after 24 hours treatment were 18.37 and 16.40, respectively. Therefore, A549 cancer cells with higher IC_50_ were more resistant to crude venom comparing to normal fibroblast cells. 

## Effect of *P. persicus* crude venom on caspase-3 and caspase-9 activities

To understand if apoptosis was the reason of cell death and to explain the pathway leading to it, the activities of caspase-3 and caspase-9 (aspartate-specific cysteine proteases) were estimated in A549 and Hu02 cell lines by substrate cleavage assay. Determination of a chromophore organic compound, which is the cleavage product of substrate DEVD-p-NA at a wavelength of 405 nm, indicates the caspase-3 enzyme activity in the cells. The same protocol but different substrate (LEHD-p-NA) was used for assaying the activity of caspase-9 enzyme. 

As shown in Figure 2B, treatment of A549 and Hu02 cell lines with the IC_50_ concentrations of *P. persicus* venom at 12 and 24 hours increased the caspase-3 and caspase-9 activities comparing untreated cells in a time-dependent manner. The activities of caspase-3 and caspase-9 peaked at 24 hours after venom treatment. However, there is a drop in their activities at 48 hours after treatment in both cell lines. The decrease in the caspase activities is probably due to the higher number of cell death after 48 hours.

These results suggest that *P. persicus* venom induces its cytotoxic effects through apoptosis in a caspase-dependent mechanism in A549 and Hu02 cell lines. 

## Fractions isolation from crude venom of *P. persicus*

**Figure 3.**
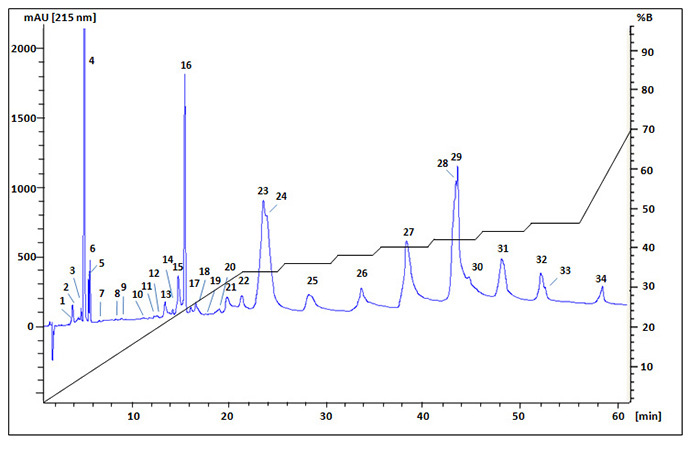
Reverse phase chromatographic separation of *P. persicus* venom. Lyophilized venom (10 mg) dissolved in 2 mL of double-distilled water was injected into C18 column continuously (100 µL of venom extract for each injection) and eluted using a semi-step gradient of acetonitrile/0.1% TFA (%B). Thirty-four protein fractions according to their peak distribution were collected manually, lyophilized and used for further analysis.

First using an analytical reverse phase HPLC (C18 column, 100 Å, 5 µm, 4.6 × 150 mm) the chromatographic profile of *P. persicus* venom components was obtained in a linear gradient of solvent B (acetonitrile/0.1% TFA). To be able to split the peaks and collect the venom components individually, the exact ratios of solvents for the peaks were estimated and a semi-step gradient elution condition was optimized. The fractions related to 34 peaks were collected manually and as it is clearly observed in Figure 3, some peaks were eluted in specific ratio of solvents. 

## Cytotoxicity analysis of HPLC fractions of *P. persicus* venom

The lyophilized fractions were dissolved in DMEM medium and A549 and Hu02 cells were treated with 20 µg/mL of each fraction for 24 hours. The cell viability was analyzed using MTT assay. 

**Figure 4.**
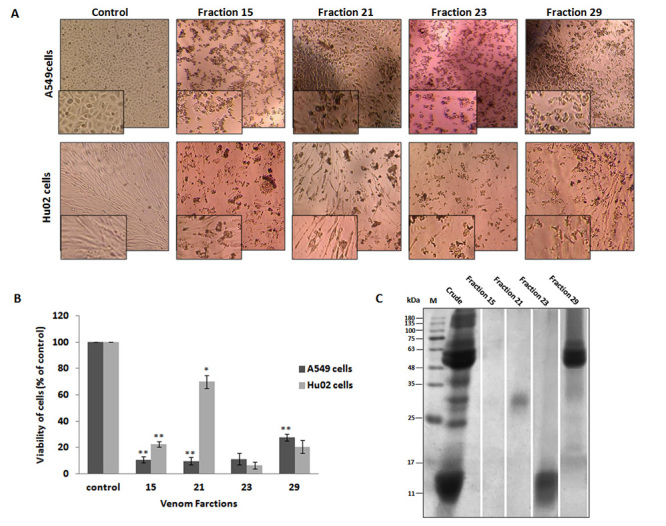
Analysis of cytotoxic fractions of *P. persicus* venom. (**A**) Morphological observation of A549 and Hu02 cells treated with HPLC fractions 15, 21, 23 and 29 under phase-contrast inverted microscope (magnification 100×). The A549 and Hu02 cells were treated with 20 µg/mL isolated fractions and incubated for 24 hours in 96-well plates. The morphological changes in the cells treated with cytotoxic fractions are displayed here. Untreated cells comprise the control group. (**B**) Effect of cytotoxic fractions of *P. persicus* venom on A549 and Hu02 cells. The cells were treated with 20 µg/mL of fractions 15, 21, 23 and 29 for 24 hours and the viability of the cells was evaluated by MTT test. Untreated cells comprise the control group. Values were presented as means ± SD (n = 3). Statistical significance versus control (*p < 0.05; **p < 0.01; ***p < 0.001). (**C**) SDS-PAGE analysis of cytotoxic fractions under reduced conditions. M: Molecular mass marker.

The fractions that reduced the survival rate of cells in less than 85% were considered to be toxic fractions. Figure 4A shows the morphological changes and the extent of cell death for A549 and Hu02 cells 24 hours after incubation with 20 µg/mL of HPLC fractions 15, 21, 23 and 29. Other fractions did not induce considerable changes in the viability and morphology of cells (data not shown). The toxic fractions exerted significant inhibitory effects on the growth rate of A549 cells. The survival rates of A549 cells following treatment with 20 µg/mL of fractions 15, 21, 23 and 29 were 10.73%, 9.57%, 11.25% and 27.50% of untreated cells, respectively. Fraction 21 exhibited the highest cytotoxicity for A549 cells and the least cytotoxicity towards Hu02 cells. The survival rates of Hu02 cells treated with 20 µg/mL of fractions 15, 21, 23 and 29 were 22.50%, 69.88%, 6.54% and 20.47% of untreated cells, respectively (Figure 4B). 

The molecular masses of the protein fractions responsible for the cytotoxic activity of venom were evaluated using SDS PAGE analysis (Figure 4C). Fraction 15 did not show any signal on the gel, which supposed to be a small peptide (< 10 kDa) or a non-protein/non-peptide compound of the venom. Fraction 21 showed a single band on SDS-PAGE analysis with a molecular mass between 25 and 35 kDa and fraction 23 contained low molecular mass peptides with molecular masses between 11 and 17 kDa. Fraction 29 contained high molecular mass proteins (between 48 and 63 kDa - Figure 4 C). Using the proteome map of *P. persicus* venom as a reference we are able to get additional information about the proteins present at the cytotoxic fractions [16]. 

## Analysis of HPLC fraction 21 of *P. persicus* venom

In order to find out if the cytotoxic effect of the fraction 21 in cancer cells is accidental because of the non-specific factors (necrosis) or if it is specific, caspase-3 and caspase-9 activities and LDH release of A549 and Hu02 cells treated with various concentrations of this fraction were estimated.

**Figure 5.**
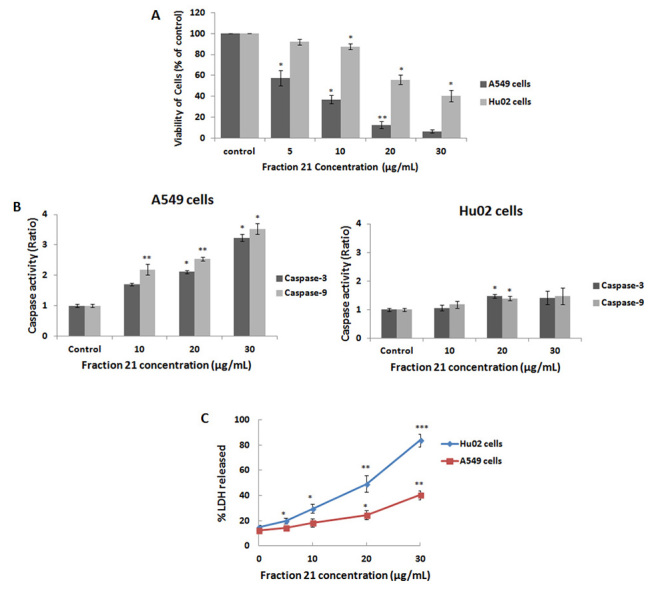
Analysis of cell viability and caspase-3 and caspase-9 activities after treatment of A549 and Hu02 cells with different concentrations of fraction 21 of *P. persicus* venom. (**A**) The dose-dependent effect of fraction 21 on the cell viability of A549 and Hu02 cell lines. (**B**) The caspase-3 and caspase-9 activities of the A549 and Hu02 cells after treatment with different concentrations of fraction 21. Relative caspase-3 and caspase-9 activities were estimated as a ratio of the cells treated with fraction 21 to untreated cell (control). (**C**) Effects of different concentrations of fraction 21 on the percentage of LDH released in A549 and Hu02 cells 24 hours after incubation. All data were presented as mean ± S.D (n = 3). Statistical significance *versus* control (*p < 0.05; **p < 0.01; ***p < 0.001).

As it is observed in Figure 5A, fraction 21 decreased the viability of A549 cells in a dose-dependent manner. By treating the cells with 30 µg/mL of this fraction, the cell viability decreased to 6.67 ± 1.2%. However, Hu02 cells were very resistant to this toxic fraction and after treatment with highest concentration (30 µg/mL) of fraction 21, the viability of these cells was 47.89 ± 2.87% compared to untreated cells. The caspase-3 and caspase-9 activities increased in A549 cells by increasing the concentration of fraction 21, but the activities of these enzymes did not change in Hu02 cells when compared to untreated cells (Figure 5B). 

Furthermore, the LDH release increased from the cells treated with different concentrations of fraction 21 in a dose-dependent manner (Figure 5C). As shown in Figure 5C, treatment of Hu02 cells with 5, 10 and 20 µg/mL of fraction 21 caused the release of 20%, 33% and 56% total LDH, respectively. However, treatment of A549 cells with the same concentrations of fraction 21, released only 14%, 18% and 24% total LDH into culture medium. Treatment of the cells with 30 µg/mL of fraction 21 caused massive release of LDH (83% of total LDH) in Hu02 cells comparing to 40% LDH release in A549 cells. 

## Discussion

The complex mixture that comprise snake venom components is responsible for numerous biological activities of venoms. Many studies on snake venom proved that some of its natural compounds have cytotoxic effects, which induce apoptosis in cancer cells by altering their cellular metabolism [1,17]. In fact, most of the drugs used for cancer treatment induce their effects through activation of essential factors of the apoptotic signaling pathways. Therefore, a compound of snake venom with ability to induce apoptosis in cancer cells is a potential candidate for an anticancer drug. Up to this moment, some drugs derived from venom peptides that inhibit the proliferation and invasion of cancer cells are already in preclinical or clinical phase studies [3]. 

The purpose of the current study was to evaluate the cytotoxicity of the *P. persicus* venom and its components on cancer and normal cells. The *in vitro* cytotoxicity test was applied to determine the anticancer activity of venom [18,19]. In this test, lung cancer cells (A549) and normal fibroblast cells (Hu02) were exposed to different concentrations of crude venom and the cell viability was estimated using MTT assay. The IC_50_ of venom for each cell line was calculated. The results showed that the crude venom significantly reduced the viability of A549 and Hu02 cells in a concentration-dependent manner. The variation in the cytotoxicity is due to the complexity of the venom components and the presence of different specific receptors on the cell surfaces [20-22]. 

Since the crude venom contains various components, cell death happens in different mechanisms of action. Although *P. persicus* crude venom has significant cytotoxic activity, since it kills normal cells (Hu02) as well as cancer cells (A549), we cannot conclude that the crude venom has anticancer activity, unless we could identify a venom component which selectively kills cancer cells with no effects on normal cells [11]. 

In order to determine the specific venom compounds that caused the cytotoxicity effects and morphological changes in cancer cells, venom components were separated using RP-HPLC. The cytotoxic potential of the *P. persicus* venom fractions were examined on A549 and Hu02 cells. Fractions 15, 23 and 29 caused a drop in the cell viability of both cancer and normal cells. However, cell treatment with 20 µL/mL of fraction 21 significantly induced cell death in A549 cancer cell with little cytotoxic effects on Hu02 normal cells. 

Our results indicate that fraction 21 increases cell death in A549 cells in a dose-dependent manner. This was accompanied by an increased level of caspase-3 and caspase-9 activities. Increased caspase-3 and caspase-9 activities indicated that the fraction 21 can exert its inhibitory effects through mitochondria dysfunction and cellular stress, leading to apoptosis. *Naja oxiana* crude venom and cardiotoxin III (CTX III) isolated from *Naja naja atra* venom induce apoptosis in cancer cells thorough this mechanism [22-24]. Treatment of A549 cells with 30 µL/mL of fraction 21 induced 40% LDH release and a 3.5 fold increase in the level of caspase-3 activity and 3.2 fold increase in the level of caspase-9 activity, which confirmed that 30 µL/mL of this fraction induces both apoptosis and necrosis in A549 cells. This phenomenon is due to the mitochondrial membrane permeability. If a toxin damages the mitochondrial inner membrane and the permeability transition pores (PTP) are formed on the membrane, this causes the loss of membrane potential and the induction of necrotic death. If the same toxin increases the mitochondrial outer membrane permeabilization (MOMP) as well, then mitochondrial outer membrane is damaged causing the release of cytochrome C and apoptosis [23]. 

The level of LDH release and caspase-3 and caspase-9 activities in Hu02 and A549 cells treated with 5, 10, 20 µL/mL of fraction 21 had pertinent effects: low caspase-3/9 activities and higher LDH release in Hu02 cells and high caspase-3/9 activities and lower LDH release in A549 cells respectively. Therefore, these concentrations of fraction 21 lead to necrosis in Hu02 cells, whereas in A549 cells the same concentrations caused apoptosis. Interestingly, 10 µg/mL of fraction 21 showed the most promising result with approximately 63% cell death in A549 cancer cells compared to the control group. The mechanism by which this fraction decreased the A549 cell proliferation is thorough caspase activation, and the increased activity of caspase-3 and caspase-9 suggests that this fraction induces its cytotoxic effects in cancer cells via intrinsic apoptotic pathway [24]. 

The *P. persicus* venom proteome map has been prepared by Ali et al. [16] using two-dimensional polyacrylamide gel electrophoresis (2D-PAGE). In order to predict the HPLC fraction 21, this protein fraction on one-dimensional SDS-PAGE and the *P. persicus* venom protein proteome map on 2D-PAGE were analyzed. 

The protein fraction 21 had a single band with molecular mass between 25 and 35 kDa. The proteins on 2D gels with similar molecular masses are SVMP (P-I), CRiSP and kallikrein [16]. CRiSPs are non-enzyme snake venom cysteine-rich secretory proteins that act as L-type Ca^2+^ and cyclic nucleotide-gated (CNG) channel-blocking toxins in some species [25-27]. No apoptotic effects for this protein have been reported so far. Therefore, CRiSP is not likely to be responsible for the cytotoxic activities observed here. Kallikrein enzymes, a subgroup of serine proteases, are involved in various biological processes such as digestion, blood coagulation, neurotransmission, and protein post-translational modifications. It was shown that crotalase - a kallikrein-like enzyme isolated from the venom of *Crotalus adamanteus* - inhibited the growth of B16 melanoma cells *in vivo* through defibrinogenation of mice, but this enzyme does not have a direct cytotoxic or cytostatic effect on cancer cells *in vitro* [28]. SVMPs are the snake venom hemorrhagic metalloproteinases that have different biological properties. They act as prothrombin activator [29], possess proteolytic activity leading to degradation of protein aggregates [30], hemorrhagic activity [31,32] and proinflammatory and apoptotic activities [33-36]. It has been proved that graminelysin I from *Trimeresurus gramineus* snake venom, a P-I SVMP, induces apoptosis in human umbilical-vein endothelial cells by degradation of extracellular matrix [37]. A P-I SVMP from *Bothrops pauloensis* snake venom (BpMP-II), also inhibited the adhesion of endothelial cells and *in vitro* angiogenesis [38]. Menaldo et al. [39] isolated a 22.9 kDa P-I metalloprotease from *Bothrops atrox* snake venom and confirmed it by RP-HPLC and SDS-PAGE.

Fraction 21 has cytotoxic and apoptotic activities and presents a molecular mass of ~25-30 kDa, that is expected to be a P-I SVMP with a proteinase domain [40]. The results of the present study indicate that this fraction in a low dose (10 µg/mL) has high cytotoxic activity against lung cancer cells. However, in higher doses it may provoke necrosis in normal fibroblast cells, which could be due to the high toxicity of this fraction for cells. 

Although the data gathered from this study is useful, it is not enough to characterize the protein. Further investigation should be performed to reveal the biochemical properties of HPLC fraction 21 and the details about its mechanism of action that leads to cell death in lung cancer cells. 

## Conclusion

Various toxins of *P. persicus* venom showed potential direct cytotoxic effects on lung cancer and normal fibroblast cells. However, fraction 21 at low concentrations displayed promising anticancer properties by inducing apoptosis in lung cancer cells. Therefore, this protein should be analyzed more extensively, since it may provide new perspectives for drug development.

### Abbreviations

2D-PAGE: two-dimensional polyacrylamide gel electrophoresis; CNG: cyclic nucleotide-gated; DMEM: Dulbecco’s modified Eagle’s medium; DMSO: dimethylsulfoxide; FBS: fetal bovine serum; IC_50_: half-maximal inhibitory concentration; LAAO: L-amino acid oxidase; LDH: lactate dehydrogenase; MOMP: mitochondrial outer membrane permeabilization; Pen-Strep: penicillin-streptomycin; PTP: permeability transition pores.
